# Effects of Additional Protein Intake on Lean Body Mass in Patients Undergoing Multimodal Treatment for Morbid Obesity

**DOI:** 10.3390/nu16060864

**Published:** 2024-03-16

**Authors:** Wiebke Stein, Helen Josephine Sauer, Nadine Oberänder, Arved Weimann, Martin Fischer

**Affiliations:** St. George Obesity Treatment Study Group, Klinikum St. Georg gGmbH, 04129 Leipzig, Germanymartin.fischer@sanktgeorg.de (M.F.)

**Keywords:** obesity, weight loss, bioelectrical impedance analysis, body composition, lean body mass, fat-free mass

## Abstract

(1) Multimodal treatment is a standard treatment for patients with obesity. However, weight loss also leads to reductions in fat-free mass. The aim was to investigate whether additional protein intake contributes to better preservation of lean body mass (LBM). (2) A total of 267 obesity patients (age 45.8 years; BMI 47.3 kg/m^2^) were included in this analysis. For the first 12 weeks of the program, patients were given a formula-based diet of 800–1000 kcal per day. Patients were divided into a control group (CG) (*n* = 148) and a protein group (PG) (*n* = 119). The PG was characterized by an additional protein intake with the aim of consuming 1.5 g of protein per kilogram of normalized body weight, whereas the CG had a protein intake of 1 g/kg/d. Bioelectrical impedance analysis was performed at the beginning (t0) and after 12 weeks (t1) of the program. (3) There were no significant differences between the groups with respect to weight loss (*p* = 0.571). LBM was also significantly reduced in both groups, without significant differences between CG and PG. (4) Increased protein intake had no significant effect on body composition of morbidly obese patients during a 12-week formula-based diet and multimodal treatment.

## 1. Introduction

Obesity is one of the most relevant diseases in society today. It has reached pandemic proportions, affecting more than one billion people worldwide [1]. Obesity is described by the World Health Organization (WHO) as an accumulation of adipose tissue that exceeds normal levels and is associated not only with increased morbidity, but also with increased mortality [2].

A BMI ≥ 40 kg/m^2^ or ≥35 with obesity-related health conditions correspond to morbid obesity [2]. The abnormal increase in fat tissue is associated with a large variety of comorbidities such as type 2 diabetes mellitus, cardiovascular diseases, cancer, and mental disorders like depression. In addition, high body weight often leads to joint problems, which can result in a lack of exercise and thus reduced energy expenditure [3].

The goals of obesity treatment are the improvement of comorbidities, the reduction in risk factors of comorbidities, the improvement of quality of life and the reduction of work absences [4]. A multimodal approach has been established as a nonsurgical treatment method for obesity, comprising nutritional, behavior, and exercise therapy [4].

As part of nutritional therapy, patients are prescribed a calorie-restricted diet as this is essential for inducing weight loss [5]. However, a problem of diet-induced weight reductions is the associated loss of functional muscle mass. In addition to functional capacity and quality of life, muscle mass is also an important determinant of basal metabolic rate. Thus, a reduction in muscle mass leads to decreases in metabolic rate [6]. In turn, a low basal metabolic rate can increase the risk of weight regain [7], which may even consist of a regain of fat rather than muscle mass [8]. A goal of nutritional therapy should therefore be to preserve as much muscle mass as possible during weight loss while maximizing the reduction in fat mass.

Changes in body composition can be detected by using bioelectrical impedance analysis (BIA). It allows quantifying fat mass and lean body mass (LBM), which includes muscle mass. Compared to other methods such as dual-energy X-ray absorptiometry (DXA), total body water estimates, computed tomography and magnetic resonance imaging (MRI), it requires little time, is simple to apply, relatively inexpensive and can be very accurate depending on the device used [9].

A diet-related factor that can affect the loss of LBM is the amount of ingested protein [10]. With respect to age-related loss of LBM, an elevated protein intake of up to 2.0 g/kg/day is recommended, which is markedly above the 0.8 g/kg/day recommended dietary allowance for adults. Adequate protein intake may also be required to prevent LBM loss associated with calorie restriction, especially when very low-calorie diets (VLCDs) are used [11]. Accordingly, calorie-restricted formula diets have been used to provide a minimum amount of protein during periods of intense calorie restrictions [12].

However, it remains unclear whether further increasing protein intake during calorie restriction can reduce the loss of LBM during weight loss. Previous studies provided inconsistent results. Some found that increased protein intake during caloric restriction helps maintain LBM compared to normal protein intake [13,14,15], especially when combined with physical activity [16,17]. However, other studies have failed to show the positive impact of additional protein intake [18,19]. Most of these studies used experimental designs and recruited rather small groups of participants with a BMI between 25 and 43 kg/m^2^. It also remains unclear as to what degree findings may translate into actual practice of weight loss treatment.

Therefore, the aim of our retrospective cohort study was to analyze real-world data from an established obesity center that offers an intensive multimodal treatment program with an initial 12-week very-low calorie diet (VLCD). At one point (i.e., in June 2018), the treatment regime was permanently altered by introducing a daily intake of additional protein powder. We wanted to compare the corresponding cohorts (i.e., the one formed prior to with the one formed after the introduction of additional protein powder) to determine whether the additional protein intake leads to a significantly higher preservation of LBM during weight reduction.

## 2. Materials and Methods

The present paper reports data from an ongoing observational study that was designed to prospectively evaluate a publicly available, nonsurgical weight loss treatment program for patients with morbid obesity. The study was conducted according to the guidelines of the Declaration of Helsinki and approved by the Ethical Review Board of the Saxonian Medical Association (protocol code EK-B-07/10-1, date 29 March 2010) [20]. It comprises a 12-month multidisciplinary lifestyle intervention with a very low-calorie diet, a 5-year follow-up care with mandatory annual checkups and a prospective evaluation. Written informed consent was taken from every patient prior to inclusion.

The primary endpoint was change in LBM after 12 weeks of therapy. As secondary analyses, subgroups were formed to break down the heterogeneous sample and to analyze the effects in individual patient groups.

### 2.1. Subjects

We analyzed data from 595 patients who participated between 2013 and 2020 in a one-year multimodality treatment program for morbid obesity. The requirements for participation were a BMI > 35 kg/m^2^ with comorbidities or a BMI > 40 kg/m^2^ and an age between 18 and 70 years. Patients with immobility, pulmonary or cardiological insufficiency and binge eating disorder, as well as female applicants who were pregnant and breastfeeding were excluded from the program. The treatment includes a formula-based low-calorie diet in the first 12 weeks.

**Figure 1:**
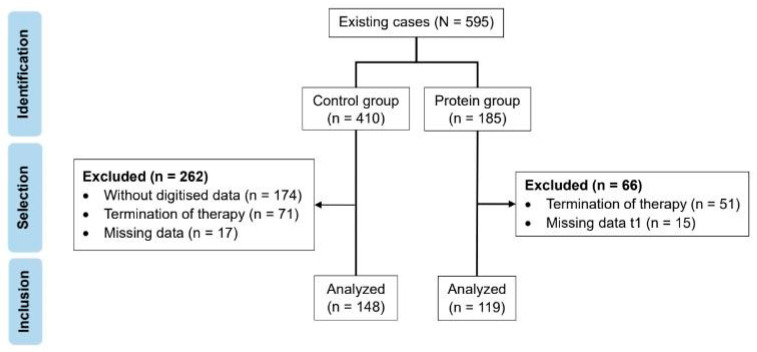
Flow chart of the study population selection.

The amount of protein provided in these first 12 weeks was increased in June 2018, making it possible to form two groups with respect to prescribed protein intake as shown in Figure 1. The allocation to the groups was based on time of participation and was not fully randomized.

The control group (CG) included 148 patients undergoing the multimodal treatment program between 2013 and 2017 and thus did not receive any additional protein supplementation.

The protein group (PG) included 119 patients who participated in our program after introduction of the additional protein supplementation, i.e., between 2018 and 2020.

### 2.2. Dietary Intervention

The intensive multimodal treatment consisted of nutritional therapy, exercise therapy and behavior therapy. A detailed description can be found in a prior publication [20].

All patients in both groups were prescribed a strict 800 kcal per day formula-based diet using OPTIFAST^®^ (Nestlé Health Science, Frankfurt/Main, Germany) as a complete meal replacement during the first six weeks. Between the 7th and 12th weeks, a period referred to as the “transition phase”, all patients were given the choice to initiate a gradual replacement of the formula-based meals with a balanced, calorie-reduced nutritional diet under the guidance of a dietitian. In week seven, patients could first add a vegetable meal with a maximum of 50 kcal to their formula diet. From the eighth week onwards, a first formula meal could be replaced by a balanced low-calorie meal. It was up to the patient to decide which of the four meals was replaced. This resulted in a daily energy intake of 950–1000 kcal.

In the control group (CG), the daily protein intake was provided solely by the consumption of the OPTIFAST^®^ products. The amount of protein was therefore predetermined by the nutritional content of these formula products and equal in all patients, regardless of their height, weight, or normalized body weight. The formula of the OPTIFAST^®^ products changed several times over the study period. On average, protein intake was around 60 g per day until 2016. Between 2016 and 2019, the daily protein intake ranged between 60 g and 80 g, depending on the combination of flavors that the patient chose from. From 2019 onwards, a new formula was introduced, providing exactly 20 g of protein among all flavors and resulting in a daily protein intake of 80 g via the OPTIFAST^®^ products. On average, the protein intake in the CG was 61.04 g, or 1.00 g per kilogram of normalized body weight at a BMI of 22 kg/m^2^ (see results).

In the protein group (PG), protein intake was individually adjusted using OPTIFAST^®^ products and additional protein powder in order to achieve a relative intake of 1.5 g per kilogram of normalized weight. Normalized weight was calculated based on a BMI of 22 kg/m^2^ for each patient. The corresponding total amount of protein was then compared with the amount provided by the formula products, and all patients were advised to substitute the remaining difference by ingesting a tasteless, sugar-free protein powder with at least 80% protein content. Patients were asked to add one to six tablespoons of protein powder to their meals throughout the day. On average, the protein intake in the PG was 92.4 g, or 1.5 g per kilogram of normalized body weight at a BMI of 22 kg/m^2^ (see results).

### 2.3. Exercise Intervention

During the first 12 weeks of our weight loss program, the sports intervention consisted of weekly instructed exercise (20 min gymnastics and 60 min endurance training). The sports therapist also prescribed a daily home exercise program (10–15 min gymnastics) and encouraged a gradual increase in daily physical activity (tracked by using pedometers).

### 2.4. Bioelectrical Impedance Analysis

Bioelectrical impedance analysis (Nutriguard-M and NutriPlus Software Version 5.3, Data Input, Pöcking, Germany) was performed according to standard protocols with Bianostic AT^®^ electrodes (Data Input, Pöcking, Germany) at the beginning and at 12 weeks of the program [21]. Assessments were scheduled in the morning and patients were asked to attend in a fasting state, wearing only light clothing and to urinate beforehand. The measurement was carried out after patients laid on their backs for about 10 min with their legs apart at an angle of approximately 45 degrees and their arms at an angle of approximately 30 degrees. Two electrodes were attached to the right hand and two to the right foot. An alternating current of 800 µA and 50 kHz was passed through the body via these electrodes. The following parameters of body composition were computed: body fat, total body water, lean body mass (LBM), extracellular mass (ECM), body cell mass (BCM), ECM/BCM, basal metabolic rate, and phase angle.

### 2.5. Additional Paramenters

Body weight was assessed in the treatment center using a scale from Kern & Sohn (Kern MTS 300K100M standing scale, minimum 2 kg, maximum 300 kg, e = 0.1 kg; Kern & Sohn GmbH, Balingen, Germany). Patients were weighed in the morning and asked to only wear light clothing and no shoes.

Body height was measured using a Seca stadiometer (Seca 206, 0–220 cm, Seca, Hamburg, Germany).

Waist/hip ratio was assessed prior to treatment initiation by trained staff adhering to standard protocols and using a Seca 201 ergonomic circumference measuring tape (Seca, Hamburg, Germany).

In patients diagnosed with type 2 diabetes mellitus (T2DM) prior to inclusion, glycated hemoglobin (HbA1c) was assessed during an in-patient stay preceding the multimodal treatment initiation.

### 2.6. Statistical Evaluation

The data were analyzed in SPSS (IBM SPSS Statistics, Version 27, IBM Deutschland GmbH, Ehningen, Germany) using the two-sample *t*-Test and the Mann–Whitney Test (continuous variables) as well as the Fisher’s Exact Test (categorical variables). Two-factor ANOVAs with repeated measures were conducted to examine changes over time between both groups. Statistical significance was assumed at *p* < 0.05.

## 3. Results

### 3.1. Baseline Characteristics

Both groups were comparable with respect to age, sex, education, implantation of gastric balloon, height, body weight and BMI. BIA-based body composition indicators, including lean body mass (LBM), also did not differ significantly between the control and the protein groups at the beginning of the program (Table 1).

The average body weight was 136 kg in the control and 132 kg in the protein group, which corresponded to a BMI of 48 kg/m^2^ and 46 kg/m^2^, respectively. The number of female participants was higher in both groups. A gastric balloon was implanted in 92 (62.2%) patients of the CG and 65 (54.6%) patients of the PG.

### 3.2. Protein Intake

In the control group, the prescribed formula-based diet provided an average daily protein intake of 61.0 g/d (SD ± 7.25, range 55–80). This corresponded to an intake of 1.0 g per kg normalized body weight at BMI 22 kg/m2 (SD ± 0.11, range 0.65–1.25).

In the protein group, each patient was prescribed a daily protein intake of 1.5 g per kg normalized body weight. The average total intake was 94.2 g/d (SD ± 11.0, range 64.7–127).

The difference in protein intake was significant between the groups (*p* < 0.001).

### 3.3. Changes in Body Weight, BMI and Lean Body Mass after 12 Weeks

After 12 weeks, body weight as well as BMI had decreased in both groups by 16% (Table 2). This difference was not significant between CG and PG.

Lean body mass (LBM) was found to be decreased significantly in both groups. The magnitude of this difference was a total of 6 kg, which corresponded to a relative loss of 8%. Statistically, the loss of LBM was found to be not significant (Table 2).

To explore whether certain groups of patients benefited from the protein supplementation, we performed several subgroup analyses with respect to sex, age, height, gastric balloon as well as baseline BMI and baseline LBM. It was found that added protein supplementation had no significant effect on LBM in men, women, older patients (i.e., age ≥ 56 years), taller patients (i.e., ≥1.74 m), patients with very high baseline BMI (≥51.6 kg/m^2^), high baseline lean body mass (i.e., ≥83.0 kg), low baseline lean body mass (i.e., ≤57.1 kg), patients with gastric balloon and patients without gastric balloon (see Supplementary Materials).

## 4. Discussion

Some studies showed that increasing protein intake during energy-restricted diets has beneficial effects on body composition and the preservation of lean body mass (LBM) [13,14,17,22,23,24]. In contrast, we found that an increased protein intake of 1.5 g as compared to 1 g per kilogram of normalized weight neither affected weight loss nor LBM loss in the course of a 12-week multimodal treatment for morbid obesity with a formula-based diet of 800–1000 kcal per day.

Indeed, there are several factors known to affect body composition during weight loss and that might help explain why the beneficial effect of increased protein could not be replicated in the present study.

One factor could be the amount of weight loss. In our study, the weight loss was 22 kg at 12 weeks. In previous studies, it ranged between 2 kg and 11 kg [14,15,16,18,25,26,27,28,29,30]. One reason for the higher loss of body weight and LBM could be the very low calorie intake (i.e., 800 kcal/d in the first 6 weeks). Larsen et al. found no significant differences in LBM between a protein and a control group in the course of a 690 kcal/d diet [31]. In their meta-analysis of very-low-calorie-ketogenic diets (VLCKDs), Muscogiuri et al. also concluded that VLCKDs which are characterized by a low carbohydrate content (<50 g/day), 1–1.5 g of protein/kg of normalized body weight, 15–30 g of fat/day and a daily intake of about 500–800 calories, do not have a better effect on conserving LBM as compared to other weight management interventions [32]. In contrast, Tang et al. recorded a significantly greater preservation of LBM in a high protein group compared with a normal protein group in the course of 2300 kcal/d [14]. This suggests that a higher calorie intake might be required to maintain LBM during weight loss intervention.

The ratio of LBM loss to total weight loss should also be considered. The amount of LBM loss was about 8% in both our study groups. In comparison, previous studies reported losses between 12 and 36% of total body weight loss [14,15,16,17,18,30,33,34]. This massively reduced LBM loss in our study could be due to multimodal treatment components such as the type and intensity of the exercise intervention. During the first 12 weeks of our weight loss program, the sports intervention consisted of 20 min/week of gymnastics exercises and 60 min/week of endurance training. In combination with the prescribed home exercise of 10–15 min daily gymnastics, it approached the WHO recommendation for physical activity of 150–300 min/week [35]. Accordingly, Layman et al. reported less LBM loss in their protein + exercise group with a minimum of 150 min/week walking +60 min/week resistance training [25]. In a study by Verreijen et al., the LBM even increased in the protein + exercise group with 180 min of resistance training per week [29]. It is thus possible that the exercise component had an effect on LBM in both our cohorts and this might have masked possible effects of protein supplementation analogous to ceiling effects in pharmacology.

Another factor is the protein intake in itself. In previous studies, control groups had a protein intake of 0.8 g/kg/d [17,24,25,29], while the control group in our analysis consumed an average of 1.0 g per day with OPTIFAST^®^ products. Ogilvie et al. found that increasing protein intake from 0.8 g to 1.0 g already had a significant effect on maintaining LBM [24]. It is unclear whether the daily intake of 1.0 g possibly represents a threshold value for protein intake, above which further increase does not provide any additional benefit [12].

Lastly, patient adherence could be a contributing factor. The formula products as well as the additional protein powder had to be financed by the patients themselves. This aspect could have had an influence on adherence to therapy. In this study, it could not be verified whether the patients followed the recommendations of protein supplementation. One characteristic that is often associated with obesity is “underreporting” [36]. This describes the underestimation or reporting of less than the actual amount of food and the associated calorie intake [36]. It is therefore possible that the reported dietary behavior of the patients during the therapy program differed from the actual food intake. Accordingly, the energy balance model would have predicted reduced weight loss in the protein group due to the calories added via additional protein supplementation (in case of adherence).

Patient adherence in the protein group could have additionally been impacted by the coronavirus pandemic as treatment had to be delivered remotely for some time [37].

A strength of our study is the real-world setting, since patients took part in an established multimodal treatment program. This allowed us to determine the alteration of a single factor (i.e., additional protein supplementation) within a complex treatment constellation involving many other factors such as motivational support, exercise and nutritional counseling. Another strength is the large study group size.

A major limitation is insufficient randomization due to the study design (i.e., observational cohort study). However, the introduction of additional protein supplementation was a fixed change to the treatment protocol and was established at a random point within the study period. Thus, the present study can be considered a form of quasi-experiment or natural study with quasi-randomization. Cohort effects have to be considered, nevertheless. The control cohort participated between 2013 and 2017 in the program and the protein cohort between 2018 and 2020. With time, therapeutic staff and techniques naturally changed and improved, respectively. Also, the program itself might have become more popular and attracted more and other patient populations. And some of our patients took part in the program during the pandemic.

Further limitations are the lack of adherence quantification and the measurement of lean body mass via BIA. Small effects on muscle mass might not be detectable by BIA [38]. Future studies should therefore focus on other measuring tools of muscle quality and function such as DXA, MRI and functional exercise tests and should carry out adherence assessments.

It also remains possible that an increased protein intake has positive effects on the body other than the preservation of muscle functioning. For example, in their analysis, Weigle et al. [39] showed that increasing protein intake from 15% to 30% of daily energy intake leads to better satiety. This also resulted in significantly lower calorie intake, significant changes in weight loss and leptin sensitivity in the central nervous system [39]. In addition, protein increases food-induced thermogenesis more than carbohydrates and fats [40] as the consumption of energy and oxygen is increased, thus increasing thermogenesis [41]. These processes also increase the feeling of satiety [42]. However, Englert et al. [18] point out in their discussion that the increase in satiety and food-induced thermogenesis due to the increase in protein intake can contribute to a higher energy deficit and thus to the loss of LBM.

## 5. Conclusions

In conclusion, our results suggest that prescribing additional protein supplementation does not help preserve lean body mass in the course of a 12-week multimodal treatment, including a formula-based VLCD that already provides a daily intake of 1.0 g.

## Figures and Tables

**Table 1:** Study population characteristics at the beginning of the program.

Variables	Control Group (CG)	Protein Group (PG)	*p*-Value ^a^
Number of patients (*n*)	148 (100%)	119 (100%)	
Age [years]	46.3 (±11.8)	45.2 (±13.1)	0.504
Sex [female:male]	101:47 (68.2:31.8%)	84:35 (70.6:29.4%)	0.691
Education [<12 y:≥12 y]	111:37 (25:75%)	81:38 (68.1:31.9%)	0.220
Gastric balloon [yes:no]	92: 56 (37.6:62.2%)	65:54 (54.6:45.4%)	0.260
Height [m]	1.67 (±0.10)	1.69 (±0.10)	0.328
Body weight [kg]	136 (±27.9)	132 (±26.8)	0.276
BMI [kg/m^2^]	48.2 (±8.02)	46.3 (±8.50)	0.070
Normalized body weight ^b^ [kg]	61.9 (±7.19)	62.8 (±7.31)	0.329
Waist–hip ratio	1.03 (±0.08)	1.04 (±0.08)	0.738
HbA1c ^c^	6.70 (5.20–13.9)	6.55 (5.50–8.80)	0.399
Body fat [kg]	66.4 (±18.3)	63.5 (±17.5)	0.198
Body fat [%]	48.8 (±7.36)	47.9 (±7.43)	0.352
Total body water [L]	51.9 (±12.9)	51.1 (±12.7)	0.641
Lean body mass (LBM) [kg]	70.8 (±17.6)	69.8 (±17.3)	0.633
Extracellular mass (ECM) [kg]	35.4 (±9.06)	35.1 (±8.53)	0.786
Body cell mass (BCM) [kg]	35.4 (±9.71)	34.7 (±9.57)	0.546
ECM/BCM	1.02 (±0.18)	1.03 (±0.16)	0.608
Basal metabolic rate [kcal]	1736 (±307)	1714 (±302)	0.552
Phase angle [°]	5.66 (±0.81)	5.57 (±0.71)	0.348

^a^: Differences were tested by using the *t*-Test (continuous variables), Mann–Whitney Test (HbA1c) or Fisher’s Exact Test (categorical variables). ^b^: Defined as body weight at BMI 22 kg/m2. ^c^: Median (Min/Max) of glycated hemoglobin (HbA1c), assessed in patients with type 2 diabetes at beginning (CG: *n* = 49, PG: *n* = 38).

**Table 2:** Changes in body weight and BMI.

		t0	t1			
		Mean (SD)	Mean (SD)	Diff.	*p*-Value (Time) ^a^	*p*-Value (Time × Group) ^b^
Body weight [kg]	CG (*n* = 148)	136 (±27.8)	113 (±24.5)	22.2 (16.4%)		
	PG (*n* = 119)	132 (±26.8)	110 (±23.5)	21.6 (16.4%)	<0.001	0.571
BMI [kg/m^2^]	CG (*n* = 148)	48.2 (±8.02)	40.3 (±7.29)	7.84 (16.3%)		
	PG (*n* = 119)	46.3 (±8.50)	38.7 (±7.93)	7.61 (16.4%)	<0.001	0.444
LBM [kg]	CG (*n* = 148)	70.8 (17.6)	65.2 (15.4)	5.68 (8.02%)		
	PG (*n* = 119)	69.8 (17.3)	64.0 (14.3)	5.79 (8.30%)	<0.001	0.860

^a^: Differences between t0 at baseline and t1 at 12 weeks. ^b^: Differences between control group (CG) and protein group (PG) at 12 weeks. Differences were tested by using the two-factor ANOVA with repeated measures.

## Data Availability

The original contributions presented in the study are included in the article and Supplementary Materials, further inquiries can be directed to the corresponding author.

## Supplementary Materials

The following supporting information can be downloaded at: https://www.mdpi.com/article/10.3390/nu16060864/s1, Table S1: Results of subgroup analyses.
