# Supplementary material for: Effects of Additional Protein Intake on Lean Body Mass in Patients Undergoing Multimodal Treatment for Morbid Obesity
Source: Nutrients. 2024 Mar 16;16(6):864. doi: 10.3390/nu16060864 (PMC10974928; doi:10.3390/nu16060864)
Supplement: Supplementary file 1 [file nutrients-16-00864-s001.zip › nutrients-2878726-supplementary.pdf]

nutrients

MDPI

Supplementary Materials

Table S1. Results of subgroup analyses.

		LBM [a] [kg] t0			LBM [kg] t1					
Subgroup		Mean (SD)	Min.	Max.	Mean (SD)	Min.	Max.	Diff.	*p*-value (time) [b]	*p*-value (time x group) [c]
Men	CG (n = 47)	91.0 (14.4)	57.9	132	82.4 (13.9)	53.0	130	8.64 (9.49 %)		
	PG (n = 35)	91.6 (15.0)	38.6	119	81.5 (12.6)	36.5	109	10.1 (11.0 %)	< 0.001	0.233
Women	CG (n = 101)	61.4 (8.86)	33.1	90.4	57.1 (7.33)	43.1	86.1	4.30 (7.00 %)		
	PG (n = 84)	60.7 (7.04)	43.9	86.3	56.7 (6.42)	40.1	79.0	3.99 (6.57 %)	< 0.001	0.555
Older patients (age 56 years) [d]	CG (n = 39)	68.0 (15.2)	51.1	107	62.5 (12.9)	47.0	101	5.54 (8.15 %)		
	PG (n = 30)	68.9 (14.6)	52.5	103	62.7 (12.0)	48.7	90.5	6.16 (8.94 %)	< 0.001	0.552
Taller patients (height ≥1.74m) [d]	CG (n = 35)	91.6 (15.6)	61.1	132	82.7 (14.6)	56.0	130	8.92 (7.00 %)		
	PG (n = 33)	85.5 (15.9)	56.5	115	77.4 (12.1)	54.6	102	8.13 (7.00 %)	< 0.001	0.577
Higher BMI (≥51.6kg/m²) [d]	CG (n = 40)	78.2 (19.9)	33.1	132	72,0 (16.7)	52.9	119	6.28 (8.03 %)		
	PG (n = 27)	79.4 (19.3)	54.6	119	72.0 (16.1)	51.3	109	7.39 (9.30 %)	< 0.001	0.485
Lower LBM (≤57.7kg) [e]	CG (n = 37)	53.4 (4.01)	33.1	57.2	51.2 (3.47)	43.1	63.2	2.22 (4.15 %)		
	PG (n = 30)	53.3 (4.27)	38.6	57.7	50.5 (4.69)	36.5	59.1	2.80 (5.26 %)	< 0.001	0.598
Higher LBM (≥83.0kg) [d]	CG (n = 40)	95.3 (11.7)	83.9	132	85.8 (12.5)	68.5	130	9.44 (9.91 %)		
	PG (n = 27)	97.0 (10.5)	83.0	119	85.7 (9.10)	67.6	109	11.3 (11.6 %)	< 0.001	0.163
With gastric balloon	CG (n = 92)	68.3 (17.5)	33.1	132	63.3 (15.5)	43.1	130	5.02 (7.32 %)		
	PG (n = 65)	71.2 (18.5)	43.9	119	65.7 (15.4)	40.1	109	5.55 (7.72 %)	< 0.001	0.523
Without gastric balloon	CG (n = 56)	75.0 (17.0)	52.0	128	68.3 (14.7)	47.2	119	6.77 (8.93 %)		
	PG (n = 54)	68.1 (15.8)	38.6	108	62.0 (12.6)	36.5	92.8	6.07 (8.96 %)	< 0.001	0.416
Combined subgroup [f]	CG (n = 95)	68.2 (16.2)	33.1	128	63,0 (13.7)	43.1	119	5.28 (7.73 %)		
	PG (n = 95)	67.4 (15.6)	43.9	119	62.2 (13.2)	40.1	109	5.14 (5.28 %)	< 0.001	0.830

a: Lean body mass; b: differences between t0 at baseline and t1 at 12 weeks; c: differences between control group (CG) and protein group (PG); d: subgroup was formed based on a 75th percentile cutoff; e: subgroup was formed based on a 25th percentile cutoff; f: Combined group consisted of 95 case-control pairs that were individually matched for sex, age (±3 years) and height (±0.05m). Differences were tested by using the two-factor ANOVA with repeated measures.
